# Intensified tuberculosis treatment to reduce the mortality of HIV-infected and uninfected patients with tuberculosis meningitis (INTENSE-TBM): study protocol for a phase III randomized controlled trial

**DOI:** 10.1186/s13063-022-06772-1

**Published:** 2022-11-08

**Authors:** Thomas Maitre, Maryline Bonnet, Alexandra Calmy, Mihaja Raberahona, Rivonirina Andry Rakotoarivelo, Niaina Rakotosamimanana, Juan Ambrosioni, José M. Miró, Pierre Debeaudrap, Conrad Muzoora, Angharad Davis, Graeme Meintjes, Sean Wasserman, Robert Wilkinson, Serge Eholié, Frédéric Ello Nogbou, Maria-Camilla Calvo-Cortes, Corine Chazallon, Vanessa Machault, Xavier Anglaret, Fabrice Bonnet

**Affiliations:** 1 Sorbonne Université, INSERM U1135, Cimi-Paris, Department of Pneumology and Thoracic oncology, Reference Centre for Rare Lung Diseases, APHP Tenon Hospital, Paris, France; 2 Université Montpellier, IRD, INSERM, TransVIHMI, Montpellier, France; 3 Division of Infectious Diseases, HIV-AIDS Unit, Geneva University Hospitals, Geneva, Switzerland; 4 Centre d’Infectiologie Charles Mérieux (CICM), Antananarivo, Madagascar; 5 University of Antananarivo, Antananarivo, Madagascar; 6 Infectious Diseases Department, University Hospital Joseph Raseta Befelatanana, Antananarivo, Madagascar; 7 Infectious Diseases Department, University Hospital Tambohobe, Fianarantsoa, Madagascar; 8 Faculty of Medicine, University of Fianarantsoa, Fianarantsoa, Madagascar; 9 Mycobacteria Unit, Institut Pasteur de Madagascar, Antananarivo, Madagascar; 10 HIV Unit, Infectious Diseases Service, Hospital Clínic-IDIBAPS, University of Barcelona, Barcelona, Spain; 11 CIBERINFEC. Instituto de Salud Carlos III, Madrid, Spain; 12 CEPED, Institut de Recherche pour le Développement, Université Paris Descartes, INSERM 1244, Paris, France; 13 Department of Internal Medicine, Mbarara University of Science and Technology, Mbarara, Uganda; 14 Médecins Sans Frontières (MSF) Epicentre, Mbarara, Uganda; 15 The Francis Crick Institute, Midland Road, NW 1AT, London, UK; 16 Faculty of Life Sciences, University College London, WC1E 6BT, London, UK; 17 Wellcome Centre for Infectious Diseases Research in Africa (CIDRI-Africa), Institute of Infectious Disease and Molecular Medicine, University of Cape Town, Cape Town, Republic of South Africa; 18 Department of Medicine, University of Cape Town, Cape Town, South Africa; 19 Division of Infectious Diseases and HIV Medicine, Groote Schuur Hospital, University of Cape Town, Cape Town, South Africa; 20 Department of Infectious Diseases, Imperial College, London, W12 0NN UK; 21 Centre Hospitalier Universitaire (CHU) Treichville, Abidjan, Ivory Coast; 22 Programme ANRS Coopération Côte d’Ivoire (PAC-CI), Abidjan, Ivory Coast; 23 Inserm – ANRS|MIE (Emerging Infectious Diseases), Paris, France; 24 University of Bordeaux, National Institute for Health and Medical Research (INSERM) UMR 1219, Research Institute for Sustainable Development (IRD) EMR 271, Bordeaux Population Health Centre, Bordeaux, France; 25 CHU de Bordeaux, Saint-André Hospital, Service de Médecine Interne et Maladies Infectieuses, 1 rue Jean Burguet, 33075 Bordeaux, Cedex France

**Keywords:** Randomized controlled trial, Tuberculous meningitis, Aspirin, Linezolid, HIV, High-dose rifampicin

## Abstract

**Background:**

Tuberculous meningitis (TBM) is the most lethal and disabling form of tuberculosis (TB), particularly in sub-Saharan Africa. Current anti-TB treatment is poorly effective since TBM mortality reaches 40% in HIV-negative patients and up to 70% in HIV-co-infected patients. To reduce TBM-induced morbidity and mortality, the INTENSE-TBM trial evaluates two interventions in both HIV-infected and uninfected patients: an anti-TB treatment intensification using oral high-dose rifampicin (35 mg/kg daily) and linezolid (1200 mg daily and then 600 mg daily) during the first 8 weeks of the anti-TB treatment and the use of adjunctive aspirin (200 mg daily).

**Methods:**

This is a randomized controlled, phase III, multicenter, 2 × 2 factorial plan superiority trial. The trial has four arms, combining the two experimental treatments (intensified TBM regimen and aspirin) with the two reference treatments (WHO standard TB treatment and placebo), and is open-label for anti-TB treatment and double-blind placebo-controlled for aspirin treatment. This trial is conducted in adults or adolescents of age ≥15 years with TBM defined as “definite,” “probable,” or “possible” using Tuberculosis Meningitis International Research Consortium criteria, in four African countries: Ivory Coast, Madagascar, Uganda, and South Africa. The primary outcome is all-cause death between inclusion and week 40.

**Discussion:**

The INTENSE-TBM trial represents a key opportunity to enhance TBM treatment with widely available existing drugs notably in high-incidence settings of both TB and HIV. The trial design is pragmatic and the results will permit early and effective applications in TBM patient care, in both HIV and TB high-incidence countries.

**Trial registration:**

ClinicalTrials.gov NCT04145258. Registered on October 30, 2019.

**Supplementary Information:**

The online version contains supplementary material available at 10.1186/s13063-022-06772-1.

## Administrative information

**Table:** 

Data category	Information
Primary registry and trial identifying number	ClinicalTrials.gov NCT04145258
Date of registration in primary registry	30 October 2019
Secondary identifying numbers	ANRS 12398 INTENSE-TBM
Source(s) of monetary or material support	European and Developing Countries Clinical Trials Partnership (EDCTP)
Primary sponsor	ANRS Emerging Infectious Diseases
Contact for public queries	VM, université de Bordeaux (vanessa.machault@u-bordeaux.fr; contact@intense-tbm.org) FE, Programme PAC-CI (frederic.ello@pac-ci.org)
Contact for scientific queries	FB, université de Bordeaux, CHU de Bordeaux (fabrice.bonnet@chu-bordeaux.fr; contact@intense-tbm.org) FE, Programme PAC-CI (frederic.ello@pac-ci.org)
Public title	Intensified Tuberculosis Treatment to Reduce the Mortality of Patients With Tuberculous Meningitis (INTENSE-TBM)
Scientific title	Intensified tuberculosis treatment to reduce the mortality of HIV-infected and uninfected patients with tuberculosis meningitis: a phase III randomized controlled trial
Countries of recruitment	Ivory Coast, Madagascar, Uganda, South Africa
Health condition(s) or problem(s) studied	Tuberculous meningitis
Intervention(s)	Drug: Aspirin Drug: Placebo of aspirin Drug: WHO TBM treatment Drug: Intensified TBM treatment
Key inclusion and exclusion criteria	Ages eligible for study: ≥15 years Sexes eligible for study: both Accepts healthy volunteers: no
	Inclusion criteria: age ≥ 15 years, TBM defined as “definite”, “probable” or “possible”, using criteria proposed by the Tuberculosis Meningitis International Research Consortium, Informed consent signed by the patient.
	Exclusion criteria: having received >5 days of TB treatment, renal failure (eGFR<30 ml/min, CKD-EPI formula), neutrophil count < 0.6 × 10^9^/L, hemoglobin concentration < 8 g/dL, platelet count < 50 × 10^9^/L, ALT > 5 times the Upper Limit of Normal, clinical evidence of liver failure or decompensated cirrhosis, for women: more than 17 weeks pregnancy or breastfeeding, for patients without decrease level of consciousness (Glasgow Coma Scale = 15): Peripheral neuropathy scoring Grade 3 on the Brief Peripheral Neuropathy Score (BPNS), documented *M.* tuberculosis resistance to rifampicin, positive gram-stain, bacterial culture or cryptococcal antigen in the cerebrospinal fluid or blood, evidence of active bleeding (hemoptysis, gastrointestinal bleeding, hematuria, intracranial bleeding), inability to collect cerebrospinal fluid, except for patients with confirmed tuberculosis (by rapid molecular test or culture) from another biological sample and clinical and/or CT scan evidence of meningitis, major surgery within the last two weeks prior to inclusion, ongoing chronic aspirin treatment (e.g., for cardiovascular risk), current use of drugs contraindicated with study drugs and that cannot be safely stopped, in available history from patients: evidence of past intracranial bleeding; evidence of past of peptic ulceration; evidence of recent (< 3 months) gastrointestinal bleeding; known hypersensitivity contraindicating the use of study drugs (aspirin, rifampicin, linezolid, isoniazid, pyrazinamide, ethambutol); evidence of porphyria, any reason which at the discretion of the investigator would compromise safety and cooperation in the trial.
Study type	Interventional
	Allocation: randomized intervention model. Masking: Quadruple (Participant, Care Provider, Investigator, Outcomes Assessor). The trial is open-label for anti-TB treatment and placebo-controlled for aspirin treatment.
	Primary purpose: treatment
	Phase III
Date of first enrolment	February 2021
Target sample size	768
Recruitment status	Recruiting
Primary outcome(s)	Rate of all-cause death [time grame: up to 40 weeks]
Key secondary outcomes	Rate of all-cause death [time frame: up to 8 weeks] Rate of all-cause death or loss to follow up [time frame: up to 40 weeks] Rate of new central neurological event or aggravation of a central neurological event existing at baseline [time Frame: up to 40 weeks] Rate of grade 3–4 adverse events (DAIDS adverse events grading table) [time frame: up to 40 weeks ]

## Background

Tuberculosis (TB) remains a global health problem concerning 8 million new cases per year and causing 1.5 million deaths in 2020 [1]. The most lethal and disabling form of TB is tuberculous meningitis (TBM) [2, 3], with an estimated 100,000 new cases occurring per year, representing around 6% of extra-pulmonary TB cases. In sub-Saharan Africa, TBM mortality reaches 40% in HIV-negative patients and up to 70% in HIV-co-infected patients [1–4]. Anti-TB treatment initiation before the onset of coma predicts TBM survival [2]. TBM treatment is based on a regimen used against pulmonary TB, which probably results in suboptimal drug levels in the cerebrospinal fluid (CSF).

TBM treatment has remained unchanged for decades despite its relatively poor efficacy. High-dose rifampicin up to 35 mg/kg has been tested in phase II trials, resulting in increased rates of sputum culture conversion by two months in pulmonary TB, with acceptable hepatic tolerance and safety [5–7]. Given its incomplete meningeal penetration, rifampicin exposure is significantly reduced at the site of the infection in TBM compared to the lung in pulmonary TB [8], and increasing the rifampicin dose could be particularly relevant in the setting of TBM. In a first small randomized clinical trial (RCT), high-dose intravenous rifampicin (13 mg/kg daily) during the two first weeks with the other drugs at standard dose, showed a close to 50% reduction in mortality of TBM patients compared to the oral WHO standard regimen [9]. This effect was not confirmed in a larger phase 3 trial using an increase in rifampicin oral dose to 15mg/kg in addition to levofloxacin, which could be explained by the relatively modest increase in orally administered rifampicin dose [10, 11]. This hypothesis is supported by the results of a recent phase 2 RCT demonstrating a trend of mortality reduction with the increase of rifampicin oral dose to 30 mg/kg for the first 4 weeks of treatment in a patient with definite TBM [11].

Linezolid is a repurposed anti-TB drug used to treat drug-resistant pulmonary TB [12, 13]. Its excellent meningeal penetration makes it a promising drug to treat TBM [14, 15]. Considering rifampicin induction of the linezolid metabolism when co-administered [16–18], high-dose linezolid (1200 mg daily) might be necessary to reach linezolid target exposure during the first weeks of treatment [19]. However, due to the risk of dose- and duration-dependent toxicity [20], it may not be possible to maintain a high linezolid dose for prolonged periods. TBM deaths and neurological sequelae are in large part due to stroke and cerebral vasculitis revealed by focal neurological deficits occurring in up to 20% of TBM patients [21].

Corticosteroids are part of the standard TBM treatment, but do not prevent neurological sequelae [22]. Aspirin is an inexpensive and widely available drug, that could prevent cerebral ischemic infarction, a common cause of neurological disability in TBM, when used at low dose [23]. One placebo-controlled trial showed a significant reduction of 3 months mortality with adjunctive aspirin (150 mg daily) [21] and another trial using two different doses (81 and 1000 mg daily) suggested a potential reduction in new infarcts and deaths in the aspirin-treated participants with microbiologically confirmed ITBM [24].

HIV prevalence in patients with tuberculosis is 36% in the African region and up to 60% in South Africa [1]. WHO recommended until recently using efavirenz in HIV-positive individuals on rifampicin [25]; dolutegravir is a recently approved integrase strand transfer inhibitor (InSTI), combining high antiretroviral potency, and a high genetic barrier to resistance. It is rolled out in most high HIV burden countries including for treatment of TB-HIV co-infected patients but has to be used at a double dose to limit the consequence of the rifampicin induction effect on dolutegravir metabolism [26]. However, data on the pharmacological interaction between high-dose rifampicin and dolutegravir, and on the risk of neurologic TB, immune reconstitution inflammatory syndrome (IRIS) in HIV-infected patients with TBM are lacking.

We are testing two interventions to reduce TBM morbidity and mortality: an anti-TB treatment intensification using high-dose rifampicin (35 mg/kg orally daily) and oral linezolid (1200 mg daily and then 600 mg daily) in addition to isoniazid, pyrazinamide and ethambutol used at standard doses during the first 8 weeks of the anti-TB treatment and the use of adjunctive of aspirin (200 mg daily).

## Methods

### Objectives

The primary objective is to assess the efficacy of two interventions to reduce mortality from TBM at week 40, in adolescents and adults: (1) intensified TBM treatment compared to WHO standard TBM treatment and (2) aspirin compared to not receiving aspirin (placebo).

The secondary objectives include efficacy comparisons earlier (week 8) and for different endpoints (all-cause mortality and loss to follow-up, early bacterial effect, neurological events, neurocognitive impairment, and disabilities) and assessment of safety. In a subset of patients, the study will describe the in vitro bactericidal activity of the anti-TB drugs combinations, describe plasma and CSF pharmacokinetics of rifampicin and linezolid, and the effect of high-dose rifampicin on the pharmacokinetics of linezolid and dolutegravir for HIV-infected patients. In HIV-infected patients, the risk of IRIS and new AIDS-defining illness will be compared between study arms and virological and immunological responses will be described.

### Study design

This is a randomized controlled, phase III, multicenter, 2 × 2 factorial plan superiority trial. The trial has four arms, combining the two experimental treatments (intensified TBM regimen and aspirin) with the two reference treatments (WHO standard TB treatment and placebo), and is open-label for anti-TB treatment and placebo-controlled double-blind for aspirin treatment.

### Study setting

This trial is conducted in referral hospitals of four African countries, which reflect having differing burdens of TB and HIV: Ivory Coast, Madagascar, Uganda, and Southern Africa. In Ivory Coast, patient recruitment is carried out at the three University Hospital Centers of Abidjan (Angré, Cocody, and Treichville), in Madagascar in two University Hospital Centers (Joseph Raseta Befelatanana Antananarivo, Tambohobe Fianarantsoa), in Uganda in two Regional Reference Hospital (Mbarara and Kabale Regional Reference Hospital), and in Southern Africa, in five hospitals in Cape Town (Kayelitsha District Hospital, Mitchells Plain Hospital, New Somerset Hospital) and Port Elizabeth (Linvingstone and PE central Hospitals, Dora Nginza Hospital). We designed a comprehensive capacity-building work package ensuring all centers had, or would acquire, the ability to conduct clinical trials and to develop a sustainable network of skilled researchers, clinical centers, and microbiology laboratories.

The trial is jointly coordinated by members of the INTENSE-TBM consortium grouping researchers and clinicians from the University of Bordeaux (France), Institut Pasteur Madagascar, Institut de Recherche pour le Développement (France), University of Cape Town (South Africa), Hospital Clinic of Barcelona - Consorsi Institut D’Investigacions Biomèdiques August Pi i Sunyer (Spain), Epicentre (Uganda), Centre d’Infectiologie Charles Mérieux, Université d’Antanarivo (Madagascar), Programme PACCI (Cote d’Ivoire) and University of Geneva (Switzerland).

### Study population and eligibility criteria

Participants are enrolled if they are adult or adolescent of age ≥15 years with TBM defined as “definite,” “probable,” or “possible” using Tuberculosis Meningitis International Research Consortium criteria [27]. Definite TBM is defined by either acid-fast bacilli (AFB) seen in CSF, positive CSF from *M. tuberculosis* culture, or *M. tuberculosis* commercial nucleic acid amplification test in the setting of symptoms suggestive of meningitis. Probable and possible TBM are defined using a modified Marais score (see Additional file 1) with a total score ≥12 points when neuroimaging is available, or 10 points when neuroimaging is not available, and at least 2 points from CSF or cerebral imaging criteria for probable TBM, and a total score of 6–11 when neuroimaging is available, or of 6-9 when neuroimaging is not available for possible TBM.

Patients are excluded if they have any of the following criteria: received >5 days of TB treatment, renal failure, neutrophil count <0.6×10^9^/L, hemoglobin concentration <8g/dL, platelet count <50×10^9^/L, ALT >5 × ULN, clinical evidence of liver failure or decompensated cirrhosis, >17 weeks pregnancy or breastfeeding for women, a peripheral neuropathy scoring grade 3 or above, documented *M. tuberculosis* resistance to rifampicin, positive cryptococcal antigen in blood or CSF, active bleeding or condition increasing the risk of bleeding, current use of drugs contraindicated with study drugs and that cannot be safely stopped, and known hypersensitivity contraindicating the use of study drugs. Inability to collect CSF is also an exclusion criterion except for patients with confirmed TB from another biological sample and clinical and/or CT scan evidence of meningitis.

### Randomization and treatment allocation

Eligible patients are randomly allocated to each arm in a 1:1:1:1 ratio. The randomization is blocked and stratified by three characteristics: Country, HIV positive/negative status, and the modified British Medical Research Council criteria (BMRC) grade of severity (2 groups: grade 1 and grade 2+3). The randomization lists were pre-established by the trial statistician and are concealed. Site investigators have permanent private access to the online randomization system, which allows them to ascertain eligibility, confirms the decision to randomize, and sequentially allocate the participant to a trial treatment.

The study is open-label for TB treatment and double-blind for aspirin and placebo administration. A table linking the randomization code with a treatment assignment was drawn centrally by an independent statistician who is the only person unblinded. The independent statistician has no other responsibilities associated with the conduct of the study and does not participate in the evaluation of adverse events or in the analysis of the data. An unblinding procedure was developed to give access to the treatment assignment for the safety of the participants in pre-established situations, such as the occurrence of blinding.

### Interventions

Anti-TB drugs and aspirin/placebo are given once a day. Whenever possible, anti-TB drugs are given orally in the morning, fasting, and aspirin/placebo with a meal. The route of administration and dose depend on the clinical assessment of patient’s ability to swallow and the presence or absence of suspected malabsorption (i.e. severe diarrhea, severe vomiting, or ileus) [27].

All patients receive the WHO standard TBM treatment using fixed dose combination with Isoniazid (5 mg/kg/day) + rifampicin (10 mg/kg/day) + ethambutol (20 mg/kg/day) + pyrazinamide (30 mg/kg) from the inclusion to end of week 8 and isoniazid (5 mg/kg/day) + rifampicin (10 mg/kg/day) from week 9 to week 40. In the Intensified TBM arm, patients receive additional doses of rifampicin to reach the total rifampicin dose of 35 mg/kg/day by oral route during the first 8 weeks and linezolid (1200 mg/d from inclusion to end of week 4 and 600 mg/day from week 5 to end of week 8). WHO body weight ranges are used for calculating the number of tablets (Table 1). Aspirin 200 mg/day (2 tablets of 100 mg) or placebo are given from the inclusion to the end of week 8.

**Table 1:** Doses and number of tablets of anti-TB drugs across the weight range

Regimen		Weight range (kg)				
		30–39	40–49	50–59	60–70	>70
Standard WHO TBM treatment	R dose calculated (Target 10 mg/kg)	350 mg	450 mg	550 mg	650 mg	>700 mg
	Fixed-dose combination R_150_ H_75_ Z_400_ E_275_	2 tablets	3 tablets	4 tablets	4 tablets	5 tablets
	R dose dispensed	300 mg	450 mg	600 mg	600 mg	750 mg
Intensified TBM treatment	R dose calculated (Target 35 mg/kg)	1225 mg	1575 mg	1925 mg	2380 mg	>2450 mg
	Fixed-dose combination R_150_ H_75_ Z_400_ E_275_	2 tablets	3 tablets	4 tablets	4 tablets	5 tablets
	LZD _600_	2 tablets^a^	2 tablets^a^	2 tablets^a^	2 tablets^a^	2 tablets^a^
	Additional R_300_	3 tablets	4 tablets	5 tablets	6 tablets	6 tablets
	R dose dispensed	1200 mg	1650 mg	2100 mg	2400 mg	2550 mg

^a^2 tablets from week 1 to week 4 then 1 tablet from week 5 to week 8

For patients unable to swallow without suspicion of malabsorption drugs are given through a naso-gastric tube at the same dose. In case of malabsorption, an intravenous infusion can be used for rifampicin, isoniazid, and linezolid at the same dose, except for rifampicin from the intensified treatment that is given at the dose of 20 mg/kg/day. The choice of this rifampicin dose is supported by the 40–50% higher bioavailability for intravenous rifampicin [28, 29]. All participants will receive dexamethasone for the first 8 weeks of TBM treatment starting at 0.4 mg/kg/day with a decrease of 0.1 mg/kg/day per week until week 4 and followed by a reduction of dose from 4 mg/d per week on week 5 at 1 mg/d per week at week 8. Gastric protection using ranitidine or proton pump inhibitors, albendazole (400 mg first 3 days) to prevent strongyloidiasis in an endemic country and pyridoxine (50 mg daily) to limit the risk of peripheral neuropathy are also given. HIV-infected patients naïve of antiretroviral therapy (ART) start this therapy 4 weeks after TB treatment using dolutegravir 50 mg twice daily (BID), with tenofovir 245 mg OD/lamivudine 300 mg OD [30]. It is not expected and overinduction of dolutegravir metabolism is induced by the higher dose of rifampicin. Study medications are dispensed by the site trial pharmacist or their delegates upon receipt of a study prescription form completed by the trial doctor. The amounts of medication dispensed and batch numbers are recorded in drug accountability logs to monitor drug dispensation. After hospital discharge, drugs are administered using directly observed treatment (DOT) by a health care worker or trained lay-provider with the possibility of community- or home-based DOT. In case of suspicion concern of adherence problems, participants will receive individual counseling by trained social workers as well as psychosocial support whenever indicated.

Standard Operating Procedures (SOPs) give guidance for the management of adverse events that can be related to study drugs such as hepatotoxicity, neurotoxicity, hematotoxicity, rash, bleeding, and hyponatremia. SOP guide for the decision to adjust the dose, interrupt or discontinue the study drug with support from a Clinical Advisory Committee (CAC) that is permanently available to support the investigators for any clinical or treatment management questions.

### Outcome measures

The primary outcome is all-cause death between inclusion and week 40. Secondary efficacy outcomes include the composite of all-cause death and loss to follow-up between day 0 and week 40, death due to TBM between day 0 and week 40, the proportion of patients depression and proportion with disability (neurocognitive impairment, functional limitations, limitation of social participation) at week 40, *M. tuberculosis* culture conversion rate, time to culture positivity and cycle threshold (GeneXpert MTB/RIF Ultra) in cerebrospinal fluid at day 7 and time to first hospital discharge. Safety outcomes include treatment-emergent grade 3 or above adverse events, according to the DAIDS adverse events (corrected version 2.1, 2017) grading tables, serious adverse events (SAE); and solicited treatment-related adverse events (between day 0 and week 40. For HIV-infected patients, additional secondary outcomes include the onset of TB-associated paradoxical IRIS between day 0 and week 40, new AIDS-defining illness between day 0 and week 40, the percentage of patients with virological success (plasma HIV-1 RNA<50 copies/mL) at week 8 and week 40 and the CD4 count change at week 28 and week 40. Pharmacological outcomes in a subset of participants include the plasma and cerebrospinal fluid pharmacokinetics of rifampicin and linezolid at day 7 and week 6 and of dolutegravir during the first month of ART. Second-line anti-TB drug susceptibility testing and in vitro time-killing curves will be performed using isolates from a subset of patients in order to determine the rate of resistance and the in vitro activity (synergy, indifference, antagonism, and bactericidal activity) of the anti-TB combinations used, respectively.

### Participant timelines

Patients presenting with presumptive tuberculous meningitis will undergo routine examination and tests and those potentially eligible are referred to the Intense-TBM trial staff for pre-inclusion in the study in order to verify study eligibility criteria. Written informed consent is obtained at pre-inclusion visit. Once the patient is enrolled and randomized, the study treatment is started as quickly as possible. Participants have scheduled visits over the 40 weeks of follow-up during which a number of clinical and paraclinical evaluations are performed and treatment prescribed, as well as unscheduled visits in case of occurrence of adverse events (Table 2). There are three visits in the first week followed up by weekly visits until week 8 and then three last visits at weeks 12, 28, and 40.

**Table 2:** Spirit figure: schedule of enrollment, interventions, and assessment

Timepoint	Study period														
	Pre-incl T−1	Incl T0	D1	D3	D7	W2	W3	W4	W5	W6	W7	W8	W12	W28	Close-out W40
**Enrolment**															
Eligibility Criteria	x														
Informed Consent	x^1^	x^2^													
Randomization		x													
**Intervention**															
Study medication		x	x	x	x	x	x	x	x	x	x	x			
Other treatments	x	x	x	x	x	x	x	x	x	x	x	x	x	x	x
Antiviral treatment ***	(x)	(x)	(x)	(x)	(x)	(x)	(x)	x	x	x	x	x	x	x	x
**Assessment**															
**Clinical assessment**															
Physical. examination*	x	x	x	x	x	x	x	x	x	x	x	x	x	x	x
Visual tests **		x	x	x	x	x	x	x	x	x	x	x	x	x	x
Neuropathy tests **	x	x	x	x	x	x	x	x	x	x	x	x	x	x	x
Functional tests **		x	x	x	x	x	x	x	x	x	x	x	x	x	x
Cognitive tests **															x
Depression tests **															x
**Imaging**															
Brain imaging	(x^3^)														
Chest X-ray	x														
**Blood test**															
HIV serology	x														
Full blood count ^†^	x			x	x	x	x	x	x	x	x	x	x	x*	x*
Plasma electrolytes ^††^	x			x	x	x	x	x	x	x	x	x	x	x*	
Creatininemia	x			x	x	x	x	x	x	x	x	x	x	x*	
Glycemia	x			x	x	x	x	x	x	x	x	x	x		
Albuminemia	x				x					x					
ALT, Bilirubin (T/U)	x			x	x	x	x	x	x	x	x	x	x	x*	x*
Cryptococcus Ag *	x*														
HIV-1 RNA Viral load *		x*						x*				x*		x*	x*
CD4 cell count *		x*						x*						x*	x*
PK rifampicin/linezolid					x					x					
**Urine tests**															
Rapid Pregnancy test	x														
Urine LAM *	x*														
**CSF**															
TBM tests ^‡^	x			(x^1^)	x			(x^2^)							
Bacterial tests ^¥^	x														
Cryptococcus Ag *	x*														
PK rifampicin/linezolid					**x**					**x**					
Sputum TB tests	x											(x^3^)			

(x^1^) Signed; (x^2^) Orally confirmed; (x^3^) As clinically indicated; (x^4^) Only when a new CSF testing is required on the basis of the CSF results at D7; (x^5^) Only for patients with positive TB tests in sputum at baseline; (x^6^) Only when some baseline tests or sample storage could not been performed at screening; (x^7^) Only for HIV-positive patients

*D* day, *W* week, *CSF* cerebrospinal fluid, *ml* milliliter, *PK-PD* pharmacokinetics-pharmacodynamics

* Includes standard neurological assessment

** In patients with normal consciousness: Visual tests: Snellen, LogMAR or Tumbling E chart to evaluate visual acuity, and 14 plates Ishihara test to screen color; Other neuropathy tests: Brief Peripheral Neuropathy Score (BPNS) and Modified Total Neuropathy Score (mTNS); Functional tests: Modified Rankin Scale and WHODAS-2 questionnaire; Cognitive tests: Deterioration Cognitive Observable [DECO] test; Depression tests: PHQ-9 questionnaire.

*** Antiretroviral treatment for HIV-infected: pre-treated patients continue ART at inclusion; ART-naive patients start ART 4 weeks after inclusion.

^**†**^ Only for 40 participants

^**††**^ Only in South Africa, for 160 participants

### Justification of sample size

We hypothesize that the cumulative probability of death among patients with TBM is 40% at 40 weeks overall (HIV-negative: 30%, HIV positive: 50%). The sample size was calculated to show that each intervention (intensified TB treatment and aspirin) could decrease mortality by at least 30% when compared to the WHO standard TBM treatment. We hypothesize that there will not be interaction between the two interventions. Based on previous studies in the same setting, we estimate that 5% of patients could be lost to follow-up at week 0. Using the sample size calculation formula for comparing survival between two strategies (log-rank test, *n* Query), an *α*-value of 5%, a 1-β power of 80%, and an inflation factor for lost to follow-up of 1.2446, the estimated sample size is 768 patients *i.e.* 192 per arm.

### Statistical analysis

The primary analysis will be done using an intention-to-treat (ITT) approach. The ITT population includes a priori all randomized patients; a patient may be excluded if he did not initiate the treatment (as long as s/he did not know to which group s/he was randomized), withdrew his/her consent or was wrongly included with respect to major eligibility criteria. A per-protocol analysis will be performed after exclusion of patients with major deviations from the protocol.

This study is a superiority trial, i.e., when comparing strategies and determining interactions, we will perform two-sided tests and use a type I error *α*-value of 5%. We will analyze the efficacy of both interventions (Intensified TBM treatment and Aspirin) to reduce the risk of reaching the primary outcome at week 40. The probability (with 95% confidence interval) of death overtime will be described using the Kaplan-Meier method. The time at risk will be the sum of at-risk follow-up days for each participant, defined as the time from the date of randomization to the date of Week-40 visit, date of death or date of the last contact with the study team. We will first use appropriate models to examine the interaction between the two interventions and to verify the proportionality of risks. If there is no significant interaction between interventions, we will use Cox proportional multivariate hazard ratio models to compare Intensified TBM treatment to WHO standard TBM treatment, and aspirin to placebo, adjusting for the initial stratification variables (trial country, HIV status, BMRC grade severity). If the proportionality of hazards assumption is not verified for one trial treatment (Intensified TBM treatment or Aspirin), results by periods of time will be presented. If the proportionality of hazards assumption is not verified for another explanatory variable, the analyses will be stratified on this variable. If there is a significant interaction between Intensified TBM treatment and Aspirin, we will stratify analyses.

Two sensitivity analyses for missing data will be used: the “missing = failure” strategy, in which any missing value will be considered as a failure and the “maximum bias” strategy, in which any missing value is considered as a failure in the strategies whose effectiveness is sought to be superior to the reference strategies and success in the other strategies.

Safety analyses will be performed on the safety population, which includes all randomized participants who had received at least one dose of study treatment. All time-dependent secondary endpoints (mortality at week 8, occurrence of serious adverse events, overall and by category, grades 3–4 adverse events, overall and by category, time to hospital discharge) will be analyzed according to the same rules as defined above for the primary endpoint.

For all categorical secondary endpoints (week 40 treatment success, week 40 disability), we will compare Intensified TBM treatment to WHO standard TBM treatment, and aspirin to placebo, using logistic or polynomial regressions, adjusting for the initial stratification variables (trial country, HIV status, BMRC grade severity) and for any other inclusion characteristics that might statistically differ between intervention groups despite randomization.

### Data management and monitoring

Data are collected on hard copy case report forms (CRF) by the clinical investigator or designated and entered by a data clerk in the database, using a centralized web-based system that is compliant with International Conference on Harmonisation (ICH) Good Clinical Practice (GCP) guidelines. The database is located on a server at Programme PAC-CI in Abidjan, Côte d’Ivoire. The trial Data Management Plan and associated SOP describe in detail the system used to computerize the data, ensure data encryption, database access, data backup, and confidentiality. All participant information is strictly confidential and information that could lead to the identification of the participant is not recorded in the study database, nor on any other paper documents or electronic files used for the study. All paper documents and electronic files needed for data management are restricted to authorized study staff, both at the international and site levels. Checks for consistency are implemented at the data entry level on site and centrally after data entry.

A trial monitoring team at international and national clinical trial units verifies according to a data monitoring plan on a regular basis that the rights, safety, and well-being of the trial participants are protected; the data recorded are accurate, complete, and consistent; the trial is conducted in accordance with the protocol, SOP, good clinical practice (GCP) and the applicable regulatory requirement(s).

Adverse events are documented and followed to adequate resolution, and SAEs are notified, within 24 hours of awareness, to the pharmacovigilance unit of the sponsor where safety data are entered in a separate database. All suspected unexpected severe adverse reactions (SUSAR) are notified by the Inserm-ANRS vigilance department to the Competent Authorities of each country concerned and to the Data Safety Monitoring Board (DSMB). In addition, the pharmacovigilance department of the sponsor prepares an annual safety report that is sent to ethical committees of each country and the DSMB.

The DSMB is composed of five experienced external experts in the field of tuberculosis, HIV infection, statistics, and pharmacology, all nominated by the trial sponsor having met before the beginning of the trial, and at least once a year thereafter to monitor the main safety and efficacy outcome measures and the overall conduct of the trial, with the aim of protecting the participants’ safety and interest.

Each site will permit authorized sponsor’s representatives, and regulatory agencies to examine (and when permitted by applicable law, to copy) clinical records for the purposes of quality assurance reviews, audits, and evaluation of the study safety, progress, and data validity.

### Committees

The Scientific Committee provides overall supervision of the trial. It ensures that the study is carried out appropriately, scientifically, clinically, and ethically.

The Trial Steering Committee is the executive body for all issues related to the trial implementation or follow-up, in accordance with the Protocol, and the Scientific Committee decisions.

The CAC is accessible on request to provide country and site investigators with advice on important decisions regarding TB treatment and ART (drug resistance, treatment failure, drug toxicity), TBM complications, management of severe IRIS, decision to discontinue study drugs and any important clinical issue for which the investigators wish to discuss before taking a decision.

### Patient and community involvement

Within the trial centers, the focus is on providing correct and comprehensive information to patients and their families and to all hospital staff.

Outside the trial centers, community focus groups including patients and community representatives can be set up to provide feedback from the community on perceptions of the study and to advise investigators on practical, social, and ethical issues arising from the conduct of the study. At the end of the trial, those groups can also communicate findings and specific messages to the patients and the broader community.

### Ethics and dissemination

The trial is being conducted in conformity with the French Public Health Code, as modified, notably, by Public Health Law no. 2004-806 of August 9, 2004, and the Law n° 2012-300 of March 5th, 2012 on research involving the human person (Jardé Law). The trial is conducted in compliance with each country’s laws and regulations, as well as with Guidelines [31, 32] for Good Clinical Practice for biomedical research on drugs for human use and ICH Good Clinical Practice E6 (R2) 09 November 2016 and European Directive no. 2005/28/EC and Good Clinical Laboratory Practice (GCLP. World Health Organization on behalf of the Special Programme for Research and Training in Tropical Diseases, 2009. The relevant ethics boards and regulatory authorities in each country approved the final version of the protocol, the information sheet, the consent form, and any other relevant trial documents before study commencement, and any amendment of these documents after the start of the study. Informed consent is signed by the participant during pre-inclusion after receiving the information on the trial objectives, duration of follow-up, potential risks, and benefits of the trial by the clinical investigator or designee. For patients with a Glasgow Coma Scale <15, the consent of a relative or a study-independent doctor is required. Deferred consent is to be obtained from the participant when their level of consciousness improves and they have the capacity to provide consent. For adolescents below the age of civil majority, the consent of at least one parent or legal guardian and the consent or assent of the adolescent is required. Separate consent is obtained for storage of biobank samples (remaining CSF, whole blood, plasma, and dried blood spot) collected at different time points of the study for potential ancillary studies. The sponsor of the study has subscribed to a civil liability insurance policy covering the risks incurred by trial participants, according to French law and international regulations. Additional consent provisions for participation in the pharmacokinetic substudy are proposed to the patient.

Transportation to and from the study center at each clinic visit; medications, tests, visits, and hospitalization, whenever they are requested or approved by the study’s medical team are free for the participants during the entire study follow-up.

The final results will be published in peer-reviewed scientific journals and will be presented at national and international conferences, as appropriate. Authorship will be defined according to the International Committee of Medical Journal Editors criteria. As stated in the patient information sheet, results will also appear on the sponsor website (https://www.anrs.fr). The datasets generated during and/or analyzed during the current study will be available from the corresponding author upon reasonable request.

The study is registered at ClinicalTrials.gov, ID: NCT04145258 and is reported according to SPIRIT guidelines (Table 2).

## Discussion

TBM remains the most lethal and disabling form of TB. The current WHO-recommended first-line treatment has a relatively poor efficacy against this extra-pulmonary location of TB, particularly among HIV-infected patients in sub-Saharan Africa [1–4]. INTENSE-TBM hypothesizes that a new treatment strategy including high-dose rifampicin and the addition of linezolid, besides a standard dose of isoniazid, pyrazinamide, and ethambutol, will reduce mortality in TBM HIV infected and uninfected patients. Furthermore, we hypothesize that aspirin, a “host-directed therapy” could also reduce mortality and limit neurological complications and disability of TBM.

Nonetheless, our study protocol may be limited by the anticipated challenges in TBM recruitment at study sites with the additional risk of heterogeneity in TBM definition between the three countries, despite the consensus Marais’ definition used. The expected recruitment was pragmatically calculated from endemic countries’ local epidemiological data, in the pre-COVID-19 pandemic period, which may overestimate the inclusion potential considering the list of non-inclusion criteria.

The current LASER-TBM phase II study (Clinicaltrial.gov NCT 03927313) is evaluating in South Africa the safety and tolerability of increased dose of rifampicin, and linezolid with adjunctive aspirin added to standard therapy for TBM in 100 patients. This study is describing the plasma and CSF pharmacokinetics of linezolid and high-dose rifampicin, the relationship between their exposures and toxicity and efficacy, and finally the effect of high-dose rifampicin on linezolid and dolutegravir exposures. Moreover, the ALTER study which is ongoing in Uganda (Clinicaltrial.gov NCT 04021121) is also providing plasma and CSF pharmacokinetic and pharmacodynamics data on rifampicin 35 mg/kg and linezolid 1200 mg daily orally. However, the INTENSE-TBM trial is the first large RCT exploring the efficacy of the combination of high-dose rifampicin and high-dose linezolid regimens in patients with TBM. Contrary to the two phase 2 ongoing studies, the INTENSE-TBM trial is powered to demonstrate a superiority in terms of mortality between the intervention and the standard of care and the use of a factorial design will allow investigators to evaluate independently each intervention. We started the phase 3 INTENSE-TBM trial before the publication of the final results provided by the two phase 2 studies considering firstly the emergency to address this major public health issue in endemic countries and secondly the encouraging pre-clinical and clinical data supporting the potential benefit of the two interventions. Outcomes criteria will allow us to assess potential benefit of the interventions on the neuro-cognitive impairment and disability at the end of treatment. HIV coinfection poses an additional challenge in TBM management because of delay in ART initiation may increase other new AIDS events and high-dose rifampicin may induce drug-drug interaction with certain ART such as dolutegravir; as previously mentioned, it is not expected reduced levels of dolutegravir compared to cases receiving a standard dose of rifampicin. The choice of four African countries, reflecting different burdens of TB and HIV, allows assessment of the impact of HIV infection and management in TBM mortality. We designed a comprehensive capacity-building work package ensuring all centers had the ability to conduct the INTENSE-TBM trial and developing a network of skilled researchers, clinical centers, and microbiology laboratories. Despite the COVID-19 pandemic, INTENSE-TBM trial initiation was achieved in all sites, promoting enhanced local healthcare systems and encouraging further clinical research [33]. Moreover, the INTENSE-TBM trial will provide additional information on pharmacokinetic challenges between high-dose rifampicin, linezolid and dolutegravir necessary to manage TBM in HIV-infected patients. Additionally, the impact of the two interventions will also be assessed on, HIV-related events such as AIDS-defining illness and paradoxical TB IRIS in HIV-infected patients and on cost-effectiveness analyses in all patients.

Even if some previous studies supported the intravenous route to increase rifampicin CSF exposure [9, 28, 34, 35], we deliberately choose to limit this route’s use to malabsorption, in order to assess the efficacy of a widely reproducible and applicable high-dose oral strategy on a worldwide basis. For the same reasons, we selected an aspirin dose widely available (200 mg daily) nearest to the one (150 mg daily) decreasing TBM mortality [21].

The INTENSE-TBM trial represents, through a strong European and African collaboration, a key opportunity to enhance TBM treatment success with widely available old drugs notably in high incidence settings of both TB and HIV. The trial design is pragmatic and results will permit early and effective applications in TBM patients’ care, which would be easy to apply in both HIV and TB high-incidence countries.

## Trial status

The protocol version number is 6.0 (30th January 2022).

**Table:** 

Version	Date	Country	Main updates
1.0	11/07/2019	International	Original
1.2^a^	05/02/2020	Madagascar	• Added: section 1.4 impacts • Modified: “patients” to “participants”
1.3	09/03/2020	Uganda	• Added: information sheet for pregnant women • Added: screening Informed Consent Form • Modified: amount of the financial compensation
3.0	11/03/2020	South Africa	• Added: specific consent process for patients in coma • Added: screening phase • Modified: ALAT inclusion criteria (ALAT<3N) • Modified: gastric protection at the discretion of the physician
1.4	12/03/2021	Uganda	• Added: 2 exclusion criteria (severe heart failure + Presence of cryptococcal antigen in the blood) • Added: recommendation to use highly effective non-hormonal contraceptive methods in the protocol and the information sheet • Modified: clarification of the informed consent collection for screening. • Modified: dosage of pyridoxine according to the SOP: 50mg OD instead of 25mg OD
5.0^a^	30/11/2020	Ivory Coast	• Added: contraception for women during TB treatment • Added: withdrawing of minors and pregnant women from the study population • Added: pregnancy test of W4 and W8 • Added: annual security report and AE list should be transmitted to regulatory authorities • Added: management of pregnancy during the first two months of the study
7.0^a^	10/02/2021	Ivory Coast	• Added: 1 exclusion criteria (Presence of cryptococcal antigen in the blood) • Modified: dosage of pyridoxine according to the SOP: 50mg OD instead of 25mg OD • Modified: possibility for a relative to consent for PK-PD sub-study
4.0	16/11/2020	South Africa	• Modified: one recruitment site
6.0	31/01/2022	South Africa	• Added: 1 exclusion criteria (Presence of cryptococcal antigen in the blood) • Modified: pyridoxine dose from 25 mg to 50 mg daily • Modified: multi-omics sub-study • Modified: possibility for a relative to consent for PK-PD sub-study
8.0^a^	15/03/2022	Ivory Coast	• Suppressed: exclusion criteria on bilirubin • Modified: gastric protection at the discretion of the physician • Modified: possibility for a relative to consent for PK-PD sub-study
9.0^a^	14/04/2022	Madagascar	• Added: 1 exclusion criteria (Presence of cryptococcal antigen in the blood) • Suppressed: exclusion criteria on bilirubin • Modified: dosage of pyridoxine according to the SOP: 50mg OD instead of 25mg OD • Modified: gastric protection at the discretion of the physician • Modified: possibility for a relative to consent for PK-PD sub-study

The study began enrollment on February 2021. The expected end of enrollment is November 2024

^a^ In French

## Supplementary Information

**Additional file 1. **Modified MARAIS Score (modified from Marais *et al*. [27]).

## Data Availability

Any data required to support can be supplied on request.

## Abbreviations

AFB: Acid-fast bacilli
AIDS: Acquired immune deficiency syndrome
ART: Antiretroviral therapy
BID: Twice daily
BMRC: British Medical Research Council
BPNS: Brief Peripheral Neuropathy Score
CAC: Clinical advisory committee
CRF: Case report form
CSF: Cerebrospinal fluid
CT: Computed tomography
DOT: Directly observed treatment
DSMB: Data Safety Monitoring Board
E: Ethambutol
EDCTP: European and Developing Countries Clinical Trials Partnership
GCP: Good clinical practices
H: Isoniazid
HIV: Human immunodeficiency virus
IRIS: Immune reconstitution inflammatory syndrome
ITT: Intention-to-treat
LZD: Linezolid
OD: Once daily
R: Rifampicin
RCT: Randomized controlled trial
SAE: Serious adverse event
SOP: Standard operating procedure
SUSAR: Suspected unexpected severe adverse reaction
TB: Tuberculosis
TBM: Tuberculous meningitis
ULN: Upper limit of normal
WHO: World Health Organization
Z: Pyrazinamide
