# Supplementary material for: Intensified tuberculosis treatment to reduce the mortality of HIV-infected and uninfected patients with tuberculosis meningitis (INTENSE-TBM): study protocol for a phase III randomized controlled trial
Source: Trials. 2022 Nov 8;23:928. doi: 10.1186/s13063-022-06772-1 (PMC9640846; doi:10.1186/s13063-022-06772-1)
Supplement: Supplementary file 1 — Additional file 1. Modified MARAIS Score (modified from Marais et al. [27]). [file 13063_2022_6772_MOESM1_ESM.pdf]

Table 3: Spirit Figure: schedule of enrollment, interventions, and assessment

Timepoint	Study period														
	**Pre-incl T-1**	**Incl T0**	**D 1**	**D 3**	**D 7**	**W 2**	**W 3**	**W 4**	**W 5**	**W 6**	**W 7**	**W 8**	**W 12**	**W 28**	**Close-out W40**
**Enrolment**															
Eligibility Criteria	x														
Informed Consent	x[1]	x[2]													
Randomization		x													
**Intervention**															
Study medication		x	x	x	x	x	x	x	x	x	x	x			
Other treatments	x	x	x	x	x	x	x	x	x	x	x	x	x	x	x
Antiviral treatment ***	(x)	(x)	(x)	(x)	(x)	(x)	(x)	x	x	x	x	x	x	x	x
**Assessment**															
**Clinical assessment**															
Physical. examination*	x	x	x	x	x	x	x	x	x	x	x	x	x	x	x
Visual tests **		x	x	x	x	x	x	x	x	x	x	x	x	x	x
Neuropathy tests **	x	x	x	x	x	x	x	x	x	x	x	x	x	x	x
Functional tests **		x	x	x	x	x	x	x	x	x	x	x	x	x	x
Cognitive tests **															x
Depression tests **															x
**Imaging**															
Brain imaging	(x[3])														
Chest X-ray	x														
**Blood test**															
HIV serology	x														
Full blood count †	x			x	x	x	x	x	x	x	x	x	x	x*	x*
Plasma electrolytes ††	x			x	x	x	x	x	x	x	x	x	x	x*	
Creatininemia †††	x			x	x	x	x	x	x	x	x	x	x	x*	
Glycemia	x			x	x	x	x	x	x	x	x	x	x		
Albuminemia	x				x					x					
ALT, Bilirubin (T/U)	x			x	x	x	x	x	x	x	x	x	x	x*	x*
Cryptococcus Ag *	x*														
HIV-1 RNA Viral load *		x*						x*				x*		x*	x*
CD4 cell count *		x*						x*						x*	x*
PK rifampicin/linezolid					x					x					
**Urine tests**															
Rapid Pregnancy test	x														
Urine LAM *	x*														
**CSF**															
TBM tests ‡	x			(x[1])	x			(x[2])							
Bacterial tests ¥	x														
Cryptococcus Ag *	x*														
PK rifampicin/linezolid					**x**					**x**					
Sputum TB tests	x											(x[3])			

D: day; W: week; CSF: cerebrospinal fluid; ml: milliliter; PK-PD: pharmacokinetics-pharmacodynamics.

* Includes standard neurological assessment

** In patients with normal consciousness: Visual tests: Snellen, LogMAR or Tumbling E chart to evaluate visual acuity, and 14 plates Ishihara test to screen color; Other neuropathy tests: Brief Peripheral Neuropathy Score (BPNS) and Modified Total Neuropathy Score (mTNS); Functional tests: Modified Rankin Scale and WHODAS-2 questionnaire; Cognitive tests: Deterioration Cognitive Observable [DECO] test; Depression tests: PHQ-9 questionnaire.

*** Antiretroviral treatment for HIV-infected: pre-treated patients continue ART at inclusion; ART-naive patients start ART 4 weeks after inclusion.

† Only for 40 participants

†† Only in South Africa, for 160 participants

(x[1]) Signed; (x[2]) Orally confirmed; (x[3]) As clinically indicated; (x[4]) Only when a new CSF testing is required on the basis of the CSF results at D7; (x[5]) Only for patients with positive TB tests in sputum at baseline; (x[6]) Only when some baseline tests or sample storage could not been performed at screening; (x[7]) Only for HIV-positive patients.

Additional files

Additional file 1: Modified MARAIS Score (modified from Marais *et al*. [27])

Criteria		
Clinical criteria (Maximum category score=6)		
–	Symptom duration of more than 5 days	**4**
–	Systemic symptoms suggestive of tuberculosis (one or more of the following): weight loss (or poor weight gain in children),night sweats, or persistent cough for more than 2 weeks	**2**
–	History of recent (within past year) close contact with an individual with pulmonary tuberculosis or a positive TST or IGRA (only in children <10 years of age)	**2**
–	Focal neurological deficit (excluding cranial nerve palsies)	**1**
–	Cranial nerve palsy	**1**
–	Altered consciousness	**1**
CSF criteria (Maximum category score=4)		
–	Clear appearance	**1**
–	Cells: 10–500 per /μl	**1**
–	Lymphocytic predominance (>50%)	**1**
–	Protein concentration greater than 1 g/L	**1**
–	CSF/plasma glucose ratio < 50% or an absolute CSF glucose concentration < 2.2 mmol/L	**1**
Cerebral imaging criteria ((Maximum category score=6)		
–	Hydrocephalus	**1**
–	Basal meningeal enhancement	**2**
–	Tuberculoma	**2**
–	Infarct	**1**
–	Pre-contrast basal hyperdensity	**2**
Evidence of tuberculosis elsewhere (Maximum category score=4)		
–	Chest radiograph suggestive of active tuberculosis: signs of tuberculosis	**2**
–	Chest radiograph suggestive of miliary tuberculosis	**4**
–	CT/ MRI/ ultrasound evidence for tuberculosis outside the CNS	**2**
–	AFB identified or *Mycobacterium tuberculosis* cultured from another source—ie, sputum, lymph node, gastric washing, urine, blood culture	**4**
–	Positive commercial *M tuberculosis* NAAT from extra-neural specimen or positive urine LAM*	**4**

* Only "NAAT from extra-neural specimen" was included in the original Marais score. We added "positive urine Lipoarabinomannan (LAM)". This alteration is in line with a consensus agreement discussed at the 3rd International Consortium meeting in Tuberculous Meningitis (Lucknow, India 2019) which concluded a revision of the existing case definition is required in order to be more reflective of current clinical practice. LAM point of care testing is now widely used in the hospitals in which we are recruiting. A recent publication

demonstrated a sensitivity of 41.8% and specificity of 90.5% making this point of care diagnostic test similar in diagnostic accuracy to culture and AFB positivity [35].

Abbreviations: AFB, acid-fast bacilli; CNS, central nervous system; CSF, cerebrospinal fluid; CT, computed tomography; IGRA, interferon gamma release assay; MRI, magnetic resonance imaging; NAAT, nucleic acid amplification test; TB, tuberculosis; TST, tuberculosis skin test; US, ultrasound
